# Assessing the Efficacy of Pixel-Level Fusion Techniques for Ultra-High-Resolution Imagery: A Case Study of BJ-3A

**DOI:** 10.3390/s24051410

**Published:** 2024-02-22

**Authors:** Yueyang Wang, Zhihui Mao, Zhining Xin, Xinyi Liu, Zhangmai Li, Yakun Dong, Lei Deng

**Affiliations:** 1 College of Resource Environment and Tourism, Capital Normal University, Beijing 100048, China; 2230902128@cnu.edu.cn (Y.W.); 2220901008@cnu.edu.cn (Z.M.); 2220902120@cnu.edu.cn (Z.X.); 2230902157@cnu.edu.cn (X.L.); 2230902112@cnu.edu.cn (Z.L.); 2230901022@cnu.edu.cn (Y.D.); 2 Key Laboratory of 3D Information Acquisition and Application, Capital Normal University, Beijing 100048, China; 3 Resource and Environmental Research Center, Chinese Academy of Fishery Sciences, Beijing 100141, China

**Keywords:** remote sensing, Beijing-3 satellite, image fusion, pan sharpening

## Abstract

Beijing Satellite 3 is a high-performance optical remote sensing satellite with a spatial resolution of 0.3–0.5 m. It can provide timely and independent ultra-high-resolution spatial big data and comprehensive spatial information application services. At present, there is no relevant research on the fusion method of BJ-3A satellite images. In many applications, high-resolution panchromatic images alone are insufficient. Therefore, it is necessary to fuse them with multispectral images that contain spectral color information. Currently, there is a lack of research on the fusion method of BJ-3A satellite images. This article explores six traditional pixel-level fusion methods (HPF, HCS, wavelet, modified-IHS, PC, and Brovey) for fusing the panchromatic image and multispectral image of the BJ-3A satellite. The fusion results were analyzed qualitatively from two aspects: spatial detail enhancement capability and spectral fidelity. Five indicators, namely mean, standard deviation, entropy, correlation coefficient, and average gradient, were used for quantitative analysis. Finally, the fusion results were comprehensively evaluated from three aspects: spectral curves of ground objects, absolute error figure, and object-oriented classification effects. The findings of the research suggest that the fusion method known as HPF is the optimum and appropriate technique for fusing panchromatic and multispectral images obtained from BJ-3A. These results can be utilized as a guide for the implementation of BJ-3A panchromatic and multispectral data fusion in real-world scenarios.

## 1. Introduction

Currently, panchromatic images with high spatial resolution are widely used in applications that require detailed spatial information. However, these images lack color information and are therefore unsuitable for resource monitoring and agricultural applications. Multispectral images, on the other hand, can provide spectral information of surface objects, but their spatial resolution is usually not high enough, which limits their practical application. Practical applications require not only high spatial resolution images but also color information. However, current sensor technology constraints make it impossible to directly obtain remote sensing images with both high spatial resolution and color information [1]. Therefore, multispectral and panchromatic image fusion is a widely used technology in remote sensing at present. It combines the spatial details of high-resolution panchromatic images with the spectral information of low-resolution multispectral images to produce high-resolution multispectral images [2,3]. The fusion process aims to combine the benefits of both types of images to improve the spatial resolution of multispectral images while minimizing loss of spectral information [4].

Image fusion is typically categorized into four levels, namely pixel level, feature level, confidence level, and decision level [5]. Specifically, pixel-level fusion involves the direct fusing of individual pixels from the original remote sensing image. It has the broadest application range and the richest research results and is also one of the current research hotspots. Fusion of multispectral images with panchromatic images is also termed pan-sharpening [6]. Many methods of pan-sharpening have been proposed over the years. In general, pan-sharpening methods can be classified into traditional component substitution methods and multi-resolution analysis methods, as well as newer model-based (MB) and deep neural network-based methods.

With the rapid development of high-resolution satellites, many remote sensing applications require both high spatial and spectral resolution. Therefore, the two must be fused into images that have both high resolution and spectral information. This significantly expands the potential applications of raw remote sensing images. Tang [7] utilized panchromatic and multispectral images from the BJ-2 and GF-2 satellites as data sources. Fusion experiments were conducted using wavelet transform, PCA (Principal Component Analysis), HPF (high-pass filter), GS (Gram–Schmidt), and Brovey fusion methods. Wu et al. [8] conducted experiments on GF-1 and ZY-3 satellite images using the GS transform, PCA transform, and CN transform fusion methods. Xing [9] chose GF-1, ZY-3, and ZY-1 02C satellite images as research objects and used five pixel-based fusion methods (PCA, Brovey, GS, IHS (Intensity-Hue-Saturation), and MT transform fusion) to conduct fusion experiments. Wang [10] chose GF-1 satellite images as the research subject and conducted fusion experiments using principal component transformation, Brovey, GS, and HSV (Hue-Saturation-Value) fusion methods. Qiu et al. [11] selected ZY-3 satellite images in Xi’an, China, as the research object, and conducted image fusion experiments using fusion methods such as Brovey, GS, and HSV. Faced with the problem that some current fusion methods have poor fusion effects on new, high-resolution remote sensing images, such as IKONOS and Quick-Bird, Zhen et al. conducted fusion experiments using traditional and improved methods and conducted a comparative analysis of the test results. To find a more suitable method for fusion of new high-resolution remote sensing images [12]. Snehmani et al. [13] used extremely high-resolution Quick Bird and WorldView-3 satellite images as data to conduct a comparative analysis of 27 different fusion methods for component substitution and multi-resolution analysis.

In recent years, model-based methods have been widely used in pan-sharpening. such as bidimensional empirical mode decomposition (BEMD) [14], tensor-based sparse modeling and hyper-Laplacian prior [15], and non-negative dictionary learning [16]. These methods aim to integrate the high-frequency components of panchromatic images into multispectral images, preserving the spectral content of the original multispectral images while improving spatial resolution.

In recent advances of image processing, deep learning has played a critical role, pan-sharpening methods have undergone new developments. In 2016, Masi et al. developed a pan-sharpening technique based on a convolutional neural network (CNN) [17]. The integration of deep learning techniques with variational models has also been explored to achieve spatial-spectral fusion, particularly for PAN/multispectral fusion scenarios [18]. Yin et al. [19] proposed a model-driven deep network for pan-sharpening.

With practice, the model-based method can produce better fusion results. However, most fusion models are built based on certain priors and assumptions. Inappropriate priors and assumptions will reduce the accuracy in model construction and result in low computational efficiency. The pan-sharpening method based on deep neural networks has higher data requirements. Good fusion results can only be achieved by using a large dataset containing a variety of images as training samples. There are also problems such as a generally long training time and difficulty in adjusting the parameters optimally. Therefore, in some cases, it may not be as efficient and effective as traditional fusion methods.

In the above research, the fusion methods of traditional component replacement method and multi-resolution decomposition method were used to fuse high-resolution remote sensing images, and the most suitable fusion method was explored. At present, there is no research on the fusion methods of the Beijing-3 satellite. It is urgent to use existing mature classical image fusion algorithms to process Beijing-3 satellite images. This paper tests six traditional pixel-level image fusion methods (HCS, HPF, modified-IHS, Wavelet, PC, and Brovey), comprehensively evaluates the fusion results, discusses appropriate fusion methods, and provides a reference for the selection of Beijing-3 data fusion methods. This has significant practical implications as it will expand their application in a variety of fields.

## 2. Materials and Methods

### 2.1. BJ-3A Data

The BJ-3A satellite was launched in June 2021. It is known for its ultra-high agility, stability, and precision. The satellite follows a sun-synchronous polar orbit with a revisit cycle of 3–5 days or less. The satellite altitude is 500 km. Beijing 3A is equipped with a high-resolution, large-width panchromatic/multispectral bi-directional camera combination. The panchromatic imaging has a resolution of 0.3–0.5 m and multispectral imaging has a resolution of 1.2–2 m. The bi-directional camera imaging width is better than 23 km. The satellite can collect nearly 250,000 square kilometers of image data every day and has the ability to carry out large-scale mapping. The main parameters are shown in Table 1.

**Figure 1:**
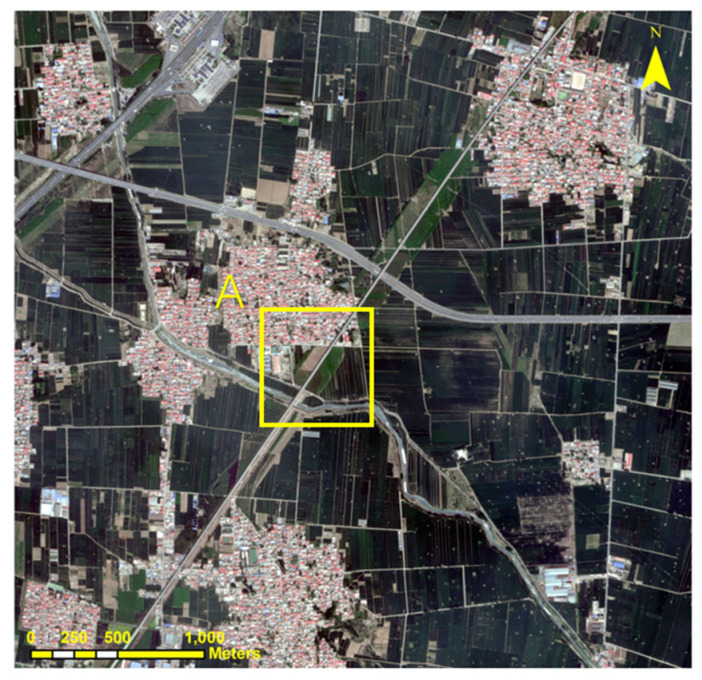
Study area view, area A will be used for subsequent local qualitative evaluation.

In this research, BJ-3A’s multispectral and panchromatic images were employed to encompass Xiong County, situated in Baoding City, within the Hebei Province of China. (Figure 1). The images were acquired on 8 May 2023. The area consists of several common surface types, including man-made buildings, farmland, vegetation, and water.

### 2.2. Data Preprocessing

The first step in processing the multispectral image involves radiometric calibration, atmospheric correction, and orthorectification. The radiometric calibration tool can be used to automatically read the correction parameters in the image header file and complete the radiation calibration. For atmospheric correction, the FLAASH model is used. The panchromatic image underwent preprocessing, including radiometric calibration and orthorectification. Finally, to ensure consistency in subsequent experiments and facilitate comparison and analysis of results, the multispectral and panchromatic images were uniformly converted to 8-bit. All of these operations were carried out using ENVI5.3.

### 2.3. Fusion Method

This study compares six frequently used pixel-level fusion methods, which are divided into two categories according to different algorithm principles: component substitution method and multi-resolution analysis method.

The component substitution methods include HCS, modified-IHS, PC, and Brovey method. The method’s core idea is to project the interpolated low-resolution multispectral image into another space using a specific conversion method. This allows for the separation of spatial structure and spectral information into different components. The spatial structure components are then replaced with panchromatic images, and the corresponding inverse transformation method generates the final high-resolution multispectral image [20].

The HCS approach involves combining high-resolution panchromatic images with lower-resolution multispectral images using the hyper spherical color sharpening algorithm. This algorithm generates a smoothed version of the panchromatic image through a sliding convolution window [21].

The IHS color system is based on visual principles and uses spatial brightness (I), hue (H), and saturation (S) as color representation systems. The RGB components of the multi-band image can be separated into the brightness, chroma, and saturation of the image after IHS transformation. To achieve this, transform the original, low-spatial resolution image from RGB space to IHS space. Then, replace the I component with the high-spatial resolution image and return to RGB space.

PCA, or principal component analysis, is a mathematical technique also known as K-L transformation. It is a multi-dimensional orthogonal linear transformation based on statistical characteristics. The PCA transformation concentrates variance information and compresses data volume, accurately revealing remote sensing information within the multi-band data structure. The primary advantage of the PCA image fusion algorithm is that it can fuse remote sensing data from more than three bands, thereby enhancing the performance of multispectral images while preserving their spectral information.

Brovey transform is also called color normalization transformation method. The principle is to first normalize the three low spatial resolution image data and then multiply them with the high spatial resolution image band.

The selected methods for multi-resolution analysis are HPF and wavelet methods. In this type of method, the high frequency components of the panchromatic image are decomposed by filters. The high frequency component is then injected into the upsampled multispectral image via the injection coefficient to obtain the fused image [22].

The principle of HPF fusion involves using a high-pass filter operator to extract structural details from high spatial resolution images. This information is then superimposed onto the low spatial resolution image to achieve fusion between the two images.

The basic principle of wavelet fusion is to decompose the original high and low spatial resolution images onto different feature domains in different frequency bands by performing wavelet positive transform, respectively. Take Mallat wavelet basis decomposition as an example. After the first layer of wavelet decomposition, the multispectral and panchromatic images are divided into four different frequency bands of sub-images composed of low–low frequency (LL), high–low frequency (HL), low–high frequency (LH), and high-frequency (HH) components, which inherit the detailed features and brightness of the original high spatial resolution image, and the spatial resolution is half of the original. For each layer of decomposed LL sub-image can be subsequently decomposed, and the number of decomposition levels depends on the features of the original image. Then, the spectral features of the multispectral image and the spatial detail features of the panchromatic image are fused at the corresponding levels, and finally the fusion result image is reconstructed using wavelet inversion.

In order to maintain the invariance of the spectral information as much as possible, when using the above fusion method for the fusion operation, the nearest neighbor method is selected as the resampling method.

### 2.4. Evaluation Method

#### 2.4.1. Qualitative Evaluation

Visual interpretation is the most common method for qualitatively evaluating remote sensing image. This method is subjective and relies heavily on the interpreter’s experience and professional knowledge. When performing visual interpretation, it is important to consider the following two aspects.

Spatial detail: evaluates whether spatial detail in the fused image is clearer and better than the original image. Evaluate whether the ground objects in the fused image are easily identifiable and more distinct than in the original image.

Spectral fidelity refers to the accuracy of colors in a fused image and their capacity to depict the spectral characteristics of ground objects.

#### 2.4.2. Quantitative Evaluation

Qualitative methods have subjective factors and are not comprehensive; therefore, they should be combined with quantitative evaluation results to compare the effects of different fusion methods, which can be achieved by analyzing different index parameters of the fusion results. In this study, five evaluation indicators were selected: mean, standard deviation, entropy [23], average gradient, and correlation coefficient [24].

The average brightness of an image is primarily described by the mean. A decent visual effect is indicated by a moderate value of mean gray. A fused image with a mean gray value that closely resembles that of the original multispectral image signifies a satisfactory fusion effect. The formula is as follows, where $\bar{Z}$ represents the average gray level, *M* and *N* are the total number of rows and columns of the image, respectively, and *i* and *j* are the pixel positions. *I* (*i*, *j*) representing the gray value of the *i*-th row and *j*-th column of the image.

$$\bar{Z}=\frac{1}{M\times N}\sum_{i=1}^{M}\sum_{j=1}^{N}I\left(i,j\right)$$

The standard deviation describes the dispersion of image grayscale relative to the mean grayscale. A larger standard deviation indicates a more dispersed grayscale distribution, resulting in higher image contrast.

$$SD=\sqrt{\frac{\sum_{i=1}^{M}\sum_{j=1}^{N}{\left[I\left(i,j\right)-\bar{Z}\right]}^{2}}{M\times N}}$$

The average gradient is a sensitive indicator of the image’s ability to depict details and describe the texture and relative clarity of the image. A larger value indicates higher image clarity.

$$\bar{G}=\frac{1}{\left(M-1)(N-1\right)}\sum_{i=1}^{M-1}\sum_{j=1}^{N-1}\sqrt{\frac{{\left[\frac{𝜕Z\left(x_{i},y_{i}\right)}{𝜕x_{i}}\right]}^{2}+{\left[\frac{𝜕Z\left(x_{i},y_{i}\right)}{𝜕y_{i}}\right]}^{2}}{2}}$$

The formula uses $\bar{G}$ to represent the average gradient, $Z\left(x_{i},y_{i}\right)$ to represent the pixel gray value of the fused image, $\frac{𝜕Z\left(x_{i},y_{i}\right)}{𝜕x_{i}}$ to represent the gradient in the horizontal direction, and $\frac{𝜕Z\left(x_{i},y_{i}\right)}{𝜕y_{i}}$ to represent the gradient in the vertical direction.

Entropy is a vital metric to evaluate the abundance and augmentation of spatial data in an image. The entropy’s magnitude reflects the average quantity of information encompassed within an image. A higher entropy value indicates a greater amount of information encapsulated within the image. A higher entropy value indicates a greater amount of information and thus, a higher quality fusion. The formula defines *E* as information entropy, $g_{i}$ as the ratio of pixels with a gray value equal to *i* to the total number of pixels in the image, and *R* as the total gray level of the image.

$$E=\sum_{i=0}^{R-1}g_{i}\log_{2}g_{i}$$

The correlation coefficient reflects the similarity of the spectral characteristics of the two images before and after fusion. The correlation coefficient indicates the degree of similarity between the fused image and the original hyperspectral image. A higher correlation coefficient suggests a closer match and higher spectral fidelity in the fused image.

$$CC=\frac{\sum_{i=1}^{M}\sum_{j=1}^{N}\left[F\left(i,j\right)-\bar{f}\right]\left[A\left(i,j\right)-\bar{a}\right]}{\sqrt{\sum_{i=1}^{M}\sum_{j=1}^{N}{\left[F\left(i,j\right)-\bar{f}\right]}^{2}{\left[A\left(i,j\right)-\bar{a}\right]}^{2}}}$$

The formula uses *F*(*i*,*j*) to represent the grayscale value of the *i*-th row and *j*-th column of the fused image. Similarly, *A*(*i*,*j*) represents the grayscale value of the *i*-th row and *j*-th column of the original multispectral image. $\bar{f}$ and $\bar{a}$ represent the mean values of the fused image and the original multispectral image, respectively.

#### 2.4.3. Comprehensive Assessment

In this study, in addition to the above evaluation indicators, spectral curves of typical ground objects, absolute errors, and evaluation methods based on object-oriented classification were added to provide a comprehensive evaluation of the fusion results.

Method 1: select typical features on the images before and after fusion, compare the differences in spectral curves between different fused images and the original images, and evaluate the degree of consistency between the spectral information of the fused images and the original multispectral images.

Method 2: spatial feature analysis is to subtract the grey values of the corresponding bands of the hyperspectral images before and after fusion and use the resulting absolute error map to evaluate the fusion effect of different bands in the spatial dimension. The formula for calculating the absolute error is as follows.

*AE_i_* = |*F_i_* − *A_i_*|

In the formula, *AE_i_* is the absolute error value of the *i*-th pixel, *F_i_* is the grey value of the *i*-th pixel in the fused image, and *A_i_* is the grey value of the *i*-th pixel corresponding to the original multispectral image.

## 3. Results and Discussion

### 3.1. Qualitative Evaluation

**Figure 2:**
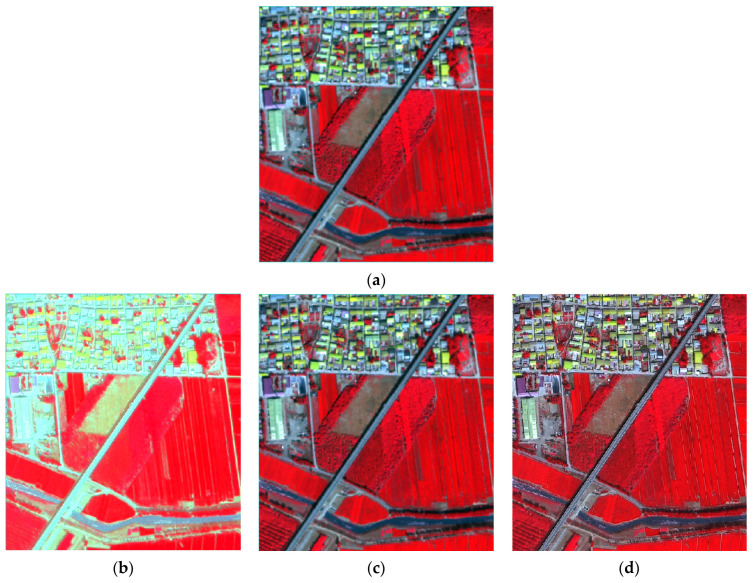

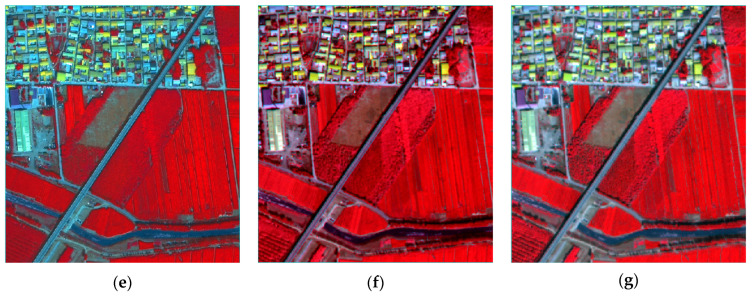
BJ-3A panchromatic and multispectral data fusion experimental results. (**a**) BJ-3A multispectral image; (**b**) Brovey image; (**c**) HCS image; (**d**) HPF image; (**e**) modified-IHS image; (**f**) PC image; (**g**) wavelet image.

Figure 2 shows the initial multispectral image of area A and the six fusion results (standard false color synthesis). In general, by comparison with the hue of the original multispectral image, except for the obvious spectral differences in the Brovey image, the outcomes achieved by other fusion methods do not significantly deviate in terms of hue. Additionally, they predominantly uphold the spectral attributes of the initial multispectral image. The HPF, wavelet, and HCS images exhibit substantial proximity to the initial multispectral image, followed by the modified-IHS and PC images, and lastly, the Brovey image.

The six fused images display the same red color for vegetation and farmland coverage areas. However, Brovey images are displayed with brighter tones. As for roads, towns, and water areas, the IHS fused image differs from the original multispectral image and appears light blue. The PC image shows darker roads, towns, and water areas, while the Brovey fused image remains brighter. The remaining fused images are comparable to the original multispectral images in terms of spectral fidelity. The HPF, HCS, and wavelet fusion methods exhibit superior performance, with almost no discernible visual differences between them. The modified-IHS and PC fused images rank second, but exhibit spectral distortion in towns, water bodies, and road areas. The Brovey fusion method performed the worst, with noticeable spectral distortion.

Due to the high resolution of the panchromatic images participating in the fusion, the fused image is required to maintain the advantage of high resolution while obtaining spectral information. This is the key to evaluating the quality of high-resolution remote sensing image fusion. Upon comparing the disparities in ground object edges and textures between the original multispectral images and various fused images, it is evident that the clarity of the other fused images has also been enhanced, except for the Brovey image. The HPF image can clearly depict the texture information of the vegetation. The boundaries of the buildings and some linear features, such as rivers and roads, are also clearly visible. The contours of the narrower paths interspersed with the farmland are also evident. This indicates that the spatial detail of the original multispectral images is greatly enhanced by the HPF fusion methods. The HCS, PC, and wavelet fused images exhibit similar performance, with average spatial details in the vegetation and farmland areas that are not significantly different from those in the original multispectral images. However, the details of buildings in urban areas have been enhanced compared to the original multispectral images, resulting in clearer edges. The modified-IHS image has a weak ability to express vegetation texture, even inferior to the original multispectral image, and there is a loss of spatial details. However, it greatly improves clarity in urban areas, and its spatial detail performance in this area is second only to the HPF image. The Brovey image loses spatial detail information, making it difficult to identify texture details of edges of buildings in towns and vegetation.

In summary, the HPF fusion method produces the best visual effect, followed by the image fused by HCS and wavelet. Both methods provide good spectral fidelity, but the HPF fusion method offers clearer spatial details of ground objects. The PC and modified-IHS fused images differ slightly from the original multispectral images and offer only average spatial detail enhancement capabilities. The Brovey fused image, however, suffers from severe spectral distortion and has the worst visual effect.

### 3.2. Quantitative Evaluation

Table 2 provides the statistical measures of the original multispectral image and six fused images, including the mean, standard deviation, entropy, average gradient, and correlation coefficient. Subsequently, this section will analyze the evaluation indicators of these six fused images.

The mean grayscale value of the HPF, HCS, and modified-IHS fused images is closest to that of the original multispectral image, followed by the wavelet fused image. However, the grayscale average of the PC fused image differs somewhat from that of the original multispectral image, and there is spectral distortion. The Brovey image exhibits a significantly lower average gray level compared to the original multispectral image. Moreover, the difference between the two is pronounced, indicating a greater degree of spectral distortion in the Brovey fused image.

The standard deviations of the wavelet, HCS, and modified-IHS images are close to those of the original multispectral images. Among them, the wavelet has the largest standard deviation at band 2, HCS has the largest standard deviation at bands 1 and 3, and modified-IHS has the largest standard deviation at band 4. The standard deviations of the HPF and PC fused images are similar to those of the original multispectral images, and slightly lower than those of the original. The Brovey image has the lowest standard deviation, indicating lower contrast and a relatively uniform tone in the image.

Except for the PC and Brovey fused images, the entropy of the images acquired through the other four fusion techniques has shown enhancement when compared to the initial multispectral image, with no significant difference in entropy. The wavelet image exhibits the greatest amount of information entropy, succeeded by the HPF and HCS fused images, with the IHS fused image being slightly lower. The above four fused images demonstrate richer information and better fusion quality. The PC and Brovey fused images contain less information than the original multispectral image.

In addition to the Brovey fused image, it can be observed that the average gradient of the image resulting from the utilization of the other five fusion methods is greater than that of the original multispectral image. This observation suggests that these five fusion methods have the capability to enhance the spatial details present in the original image. The HPF fused image has a higher average gradient and the best spatial detail enhancement effect, which is consistent with the results of visual interpretation. Additionally, the HPF fused image has the highest average gradient among the four bands, surpassing the other fused images. The second-best option is the wavelet fused image, but its average gradient in band 4 is lower than that of the modified-IHS and HCS fused images. PC, modified-IHS, and HCS fused images were compared, and there was little difference in the average gradient among the three. The Brovey image has the lowest average gradient, indicating a lower definition of the fused image.

Consistent with the results of visual interpretation, the wavelet and HCS fused images exhibit the highest correlation coefficients in bands 2, 3, and 4, indicating excellent spectral fidelity performance. Although the PC and HPF fused images do not have the highest correlation coefficients, each band still exhibits a correlation coefficient above 0.9. The correlation coefficient of modified-IHS and Brovey fused images in the near-infrared band (band 4) is lower than that of other fused images, at around 0.85. The study area comprises a significant amount of vegetation and farmland, which is more sensitive to the near-infrared band. The low correlation coefficient of the two fused images in the near-infrared band is also consistent with the visual difference from the original multispectral image in vegetated farmland areas.

### 3.3. Evaluation Based on Spectral Curves of Ground Objects

**Figure 3:**
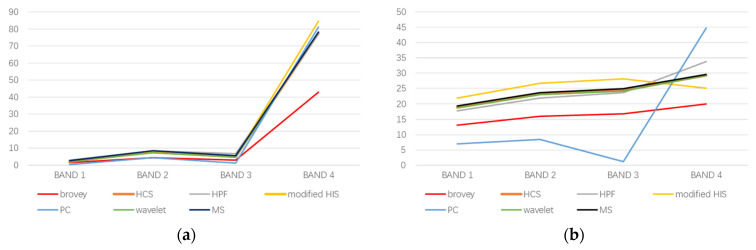

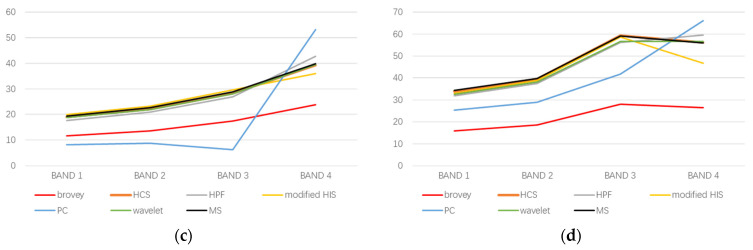
Spectral curves of ground objects from the original multispectral image and six fused images. (**a**) Vegetation; (**b**) water; (**c**) roads; (**d**) buildings.

In order to assess the spectral accuracy of diverse fused images, this study examines the disparities among the six fusion outcomes and the initial multispectral images by showcasing typical land objects including vegetation, water bodies, roadways, and buildings. In area A, we selected four types of ground objects and averaged their spectral curves over the homogeneous area. Figure 3 shows the spectral curve.

In band 4, the spectral curve of vegetation is slightly higher in the original multispectral image than in the modified-IHS fused image, while the Brovey fused image shows a significantly lower value. The other five fused images are generally in line with the original multispectral image.

With regard to the spectral curve of the water, the HCS fused image is almost identical to that of the original multispectral image. Similarly, the wavelet fused image also closely matches that of the original multispectral image. However, the HPF fused image is slightly higher than that of the original multispectral image in band 4. The modified-IHS fused image is slightly higher than that of the original multispectral image in bands 1–3, and slightly lower in band 4. The Brovey and PC fused images differ significantly from those of the original multispectral images. The Brovey fused images follow a similar trend to the original multispectral images, but the values of each band are lower. In contrast, the PC fused image differs significantly from the original multispectral image in both trend and band values, which is also reflected in the visual results.

The road exhibits a spectral curve that bears resemblance to that of the water body. Among the fused images, the HCS and wavelet demonstrate the highest degree of overlap with the fusion curves of the initial multispectral images. Correspondingly, the modified-IHS and HPF images also share similarities with the original multispectral images. In the case of the Brovey fused image, its spectral curve maintains a consistent trend with the initial multispectral image. Nevertheless, the Brovey fused image displays lower values for each band in comparison to the original multispectral image. Pertaining to the PC image, its curve showcases the most substantial discrepancy when compared to the original multispectral image, a finding that aligns with the visual outcomes.

Regarding the spectral curve analysis of the building, the HCS image exhibits the highest level of consistency with the original multispectral image. However, there are slight differences between the spectral curves of the wavelet and HPF images, compared to the original multispectral image. Notably, the modified IHS image aligns with the original multispectral image in bands 1–3 but demonstrates a lower value in band 4. Conversely, the spectral curves of both the Brovey and PC fused images significantly deviate from those of the original multispectral images. As for the PC images, bands 1–3 show a similar trend to the original multispectral images, albeit with reduced values. However, in band 4, the value is higher than that of the original multispectral image. While the spectral curve trend of the Brovey image mirrors that of the original multispectral image, the values in each band are lower.

To summarize, the fusion methods that yield the best performance are HCS, wavelet, and HPF. They are followed by the modified-IHS fusion method, and lastly, the PC and Brovey fusion method. The modified IHS image differs from the original multispectral image in band 4, similar to the visual interpretation and quantitative analysis results. In areas with vegetation, the modified IHS image differs visually from the original multispectral image, and the correlation coefficient of this band is lower than other fusion methods. The spectral curves of roads, buildings, and water in the PC fused images are significantly different from those in the original multispectral images. Differences between the PC fused images and the original multispectral images of the three types of objects are visually apparent. The spectral curve tendency of the Brovey image on the four ground objects is close to that of the original multispectral image, but there are significant differences in values, resulting in poor spectral fidelity. This is consistent with the visual and quantitative evaluation results.

### 3.4. Evaluation Based on Absolute Error

**Figure 4:**
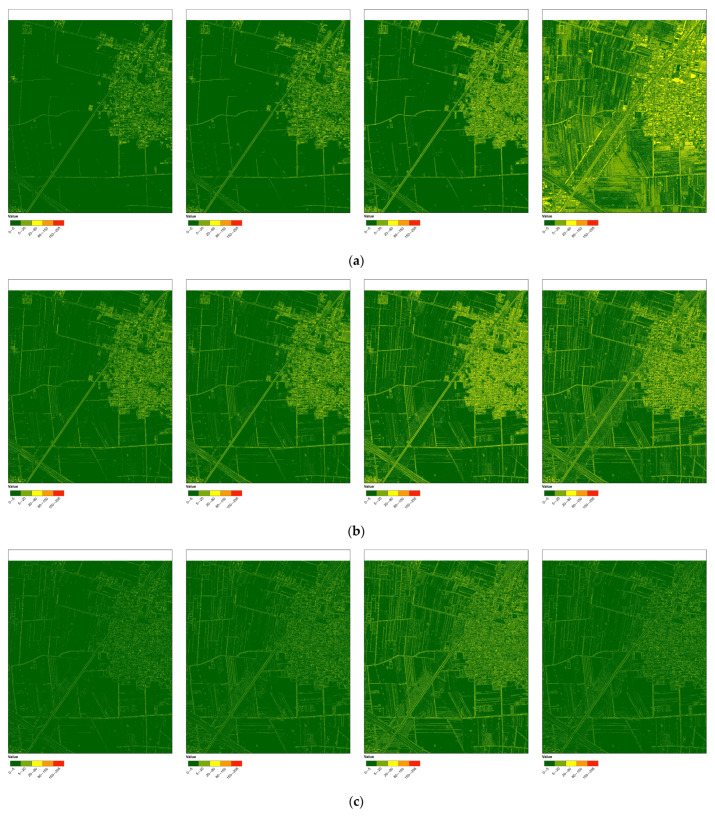

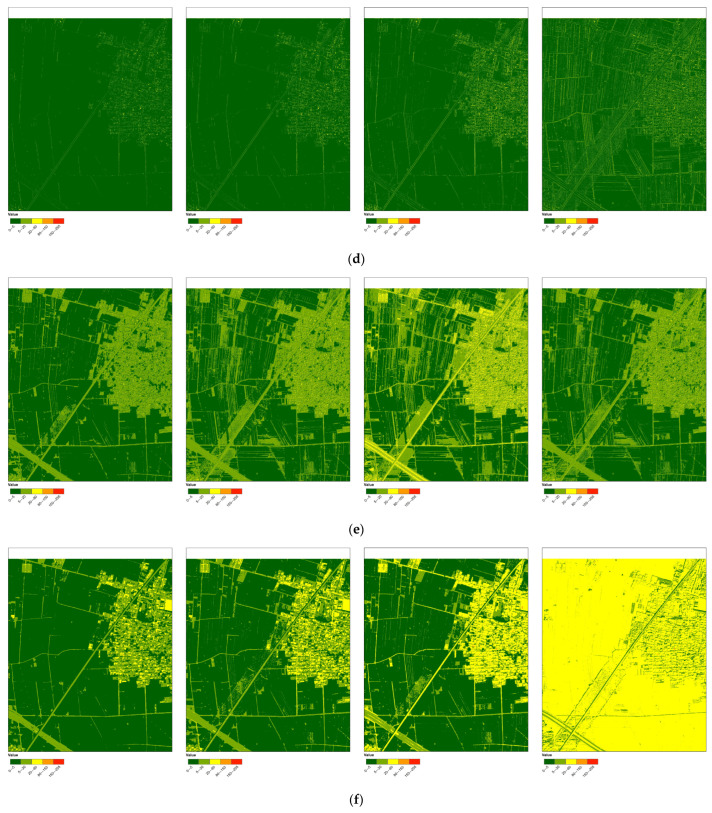
The figure displays the absolute errors of six fused images, with bands 1 to 4 arranged from left to right. (**a**) Modified-IHS; (**b**) HPF; (**c**) wavelet; (**d**) HCS; (**e**) PC; (**f**) Brovey.

The absolute error figure is generated by subtracting the absolute value of the grey scale value of the corresponding band in the fused image from the original multispectral image. In the absolute error chart of each band, for homogeneous areas, the smaller the value, the better the spectral fidelity. for the non-homogeneous areas, the larger the value, the greater the spatial detail enhancement capability. Table 3 shows the average absolute error of each band for the six fusion methods. Figure 4 displays a section of the absolute error chart for the six fused images. The absolute error values in the table and the visual effects of the homogeneous and non-homogeneous areas in the figure will be used to evaluate the fusion effects of the six fusion methods.

In general, the average absolute error of the HCS fusion images in each band is small, ranging from 1 to 2. This fusion method exhibits the best spectral fidelity, followed by the HPF and modified-IHS fusion methods, and finally the PC and Brovey. The average absolute error of the modified-IHS fusion image in the first three bands is similar to that of the wavelet fusion image (bands 1–3). However, in band 4, the error is higher, indicating slightly worse spectral fidelity performance. The average absolute error of the HPF fusion image in all four bands (bands 2–5) is slightly higher than that of the other fusion images, but it still maintains good spectral fidelity. The average absolute error value of each band in the PC fusion image is higher than that of the modified-IHS fusion image, except for band 4, which is lower. The overall spectral retention ability is average. The average absolute error of the Brovey fusion image in the first three bands is similar to that of the PC fusion image, but in band 4, it is as high as 35, indicating poor spectral fidelity.

The figure displays the absolute error of the four bands in the modified IHS fused image. In homogeneous areas, such as vegetation and farmland, the first three bands have low absolute error values, resulting in a dark green appearance, indicating better spectral fidelity. In the homogeneous area of the fourth band, a small portion appears in dark green, while the majority appears in light green and yellow. The absolute error value is higher than that of the first three bands, indicating that the spectral fidelity of this fusion method in the fourth band is not optimal. The first three bands allow for easy identification of buildings, roads, and farmland due to the yellow appearance of their edges. However, the range of these features in the fourth band is less distinct.

There is little difference in the absolute error figure among the four bands of the HPF fused image. The absolute error values are low for homogeneous areas such as vegetation, farmland, and roads, appearing as dark green. The outlines of buildings, roads and farmland as well as the textures of vegetation in the picture are also more obvious and appear yellow, making them easy to identify and the spatial details have been improved.

In the wavelet image, the absolute error figure of the four bands is represented by dark green in the homogeneous area. In the non-homogeneous area, the boundaries of different features are represented by light green and yellow. The outlines of roads, farmland, and buildings are clearer, and the spatial details of the third band are stronger than those of the other three bands. Additionally, the textures of paths in farmland and vegetation in some areas are also visible.

The absolute error figure of the HCS fused image shows that homogeneous areas appear dark green in all four bands, indicating excellent spectral preservation, but the edges of different ground objects are discontinuous in the first three bands. However, the edges of different ground objects in the first three bands are discontinuous, making it difficult to distinguish the extent of farmland and the outline of the road. Although the fourth band maintains good spectral fidelity, it also enhances spatial details, revealing farmland and road outlines, and making building edges more obvious than in the previous bands.

The absolute error figure of the four bands of the PC fused image shows little difference in each band. Vegetation and farmland areas appear as dark green, while roads and urban areas are light green in band 1, 2, and 4. Although roads, farmland, and town edges are not immediately apparent, they remain distinguishable. This is due to the spectral fidelity of PC fused images being less accurate in roads and urban areas compared to vegetation. It is this spectral difference that allows for easy differentiation.

The absolute error values of vegetation and farmland areas in the first three bands are low, appearing mainly as dark green, while roads and urban areas appear yellow in the absolute error figure of the Brovey fused image. The spectral retention ability is stronger in vegetation and farmland than in roads and urban areas, similar to PC fused images. The fourth band is predominantly yellow, indicating that the spectral preservation ability of the Brovey fusion method in this band is inferior to that of other fusion methods. In the non-homogeneous area, only major roads and the village perimeter are discernible. The smaller roads within the farmland, the texture details of the vegetation, and the outlines of the buildings in the village are almost indiscernible, indicating poor spatial detail enhancement ability.

### 3.5. Evaluation Based on Object-Oriented Classification

Image segmentation involves grouping pixels with similar characteristics to form geographical objects that reflect specific features. This process helps in the interpretation of remote sensing images and enables feature extraction, classification, target detection, and identification. For the classification of ground objects in high-resolution images, the information expressed by a single pixel becomes limited as the spatial resolution increases. This limitation makes it difficult to extract meaningful information.

High-resolution images are more suitable for image segmentation than medium- and low-resolution images because they can capture more details of ground objects due to their higher spatial resolution. On the other hand, mid- and low-resolution images have limited ability to express details, making it difficult to accurately depict small-scale features, and they are prone to producing erroneous or inaccurate segmentation results.

After fusion of multispectral and panchromatic imagery, the spatial resolution of the BJ-3A satellite imagery used in this study is 0.5 m. Therefore, due to its high spatial resolution and application, each fusion result can be segmented and classified. By analyzing the segmentation and classification results, different fusion methods can be evaluated. The e-cognition software performs image segmentation on different fusion results using the following parameters: a segmentation coefficient of 45, a shape factor of 0.4, and a compactness of 0.2.

**Figure 5:**
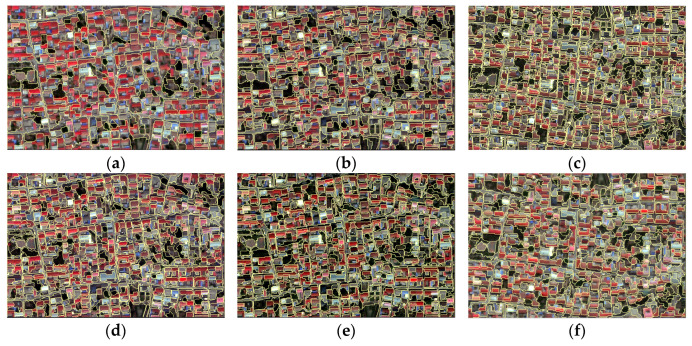
Segmentation results of 6 fused images in the village area. (**a**) Brovey; (**b**) HCS; (**c**) HPF; (**d**) modified-IHS; (**e**) PC; (**f**) wavelet.

Different fusion methods produced different segmentation results even when the same segmentation coefficient was used. The segmentation coefficient was set to be the same for the same area. The image produced by the fusion method that can better retain detailed information will have more objects after segmentation. Table 4 displays the number of objects obtained after segmenting each fused image. The Brovey fused image has the fewest number of objects. The Figure 5 reveals that, in comparison to other fused images, the Brovey fused image is under-segmented in some areas, resulting in multiple and roads being grouped into a single object. The wavelet fused image comes in second. There is little difference in the number of objects in HIS, HCS, and PC fused images. The HPF fused image has the largest number of objects.

Based on the segmentation results, we conducted object-oriented classification according to buildings, vegetation, and buildings. After selecting a sufficient number of samples, the following classification features are selected to participate in the classification: mean value of each band, NDVI, aspect ratio of the segmented object, density factor, shape index and GLCM Dissimilarity, and SVM is selected as the classifier. The overall classification accuracy and Kappa coefficient of the six fusion methods are shown in Table 5.

The segmentation and classification results suggest that HPF is more effective in preserving spatial information, resulting in clear boundaries and rich textures. As a result, the number of objects after image segmentation is greater, leading to the best overall classification effect, with the highest overall classification accuracy and Kappa coefficient. The Modified-IHS, HCS, and PC fusion methods also demonstrate superior spatial detail enhancement capabilities. The wavelet fusion method is marginally less effective than the methods mentioned above. The Brovey fusion method performs poorly, which is in line with the results obtained by other evaluation methods mentioned previously.

The study found that the HPF fusion method outperformed other fusion methods in all aspects, while the Brovey fusion method performed the worst. This is in line with the findings of the fusion experiments carried out by Wang [25] using Ziyu-3 images, Shao [26] using the Gaofen-1 satellite, and Xue [27] using the Gaofen-2 satellite. When the Brovey fusion method is applied to the above high-resolution satellites, the overall performance is poor. Other methods perform differently in different areas and using different images as research objects.

## 4. Conclusions

This article employs six fusion methods, namely modified-IHS, HPF, PC, Brovey, wavelet, and HCS, to fuse BJ-3A’s multispectral images and panchromatic images. The fusion results were evaluated comprehensively using qualitative and quantitative methods, including ground object spectral curves, absolute error figure, and object-oriented classification. The evaluation led to the following conclusions.

The HPF fusion method produces a fused image with good spectral fidelity and high definition, displaying excellent spatial details of different ground objects. This method is particularly suitable for fusing BJ-3A satellite panchromatic and multispectral images. Although the wavelet and HCS fusion methods have high spectral fidelity and improved information content compared with the original multispectral image, the HPF fusion method outperforms them in terms of spatial detail expression. The performance of the modified-IHS and PC fusion methods is moderate. The Brovey fusion method is inferior to the above five methods in terms of expressing spatial details and has relatively serious spectral distortion problems.

Except for the Brovey fusion method, which yields relatively poor results, all other methods can produce high-quality fusion results for high-resolution remote sensing images. This suggests that the simple and easy-to-implement pixel-level fusion method remains suitable for current, newer, and higher-resolution satellite images. However, it is important to note that diverse fusion methods may produce varying outcomes on different study areas. In practical applications, it is important to choose suitable data fusion methods based on specific needs.

## Figures and Tables

**Table 1:** BJ-3A satellite parameters.

Name	Index
Image resolution	PAN: 0.3–0.5 m MS: 1.2–2 m
Band range	PAN: 0.45 μm–0.70 μm Band 1 Blue: 0.45–0.52 μm Band 2 Green: 0.52–0.59 μm Band 3 Red: 0.63–0.69 μm Band 4 Near-infrared: 0.77–0.89 μm
Width	23.5 km
Incidence Angle	±45°
Descending node time	11:00 AM
Satellite capacity	Shooting capacity: 500,000 square meters/day storage: 6 TB Data transmission: 900 Mbps

**Table 2:** Quantitative evaluation indicators.

Number of Bands	Fusion Method	Mean	Standard Deviation	Entropy	Mean Gradient	Correlation Coefficient
Band 1	Original MS	9.867	11.121	4.267	5.105	
	Modified-IHS	9.516	10.794	4.246	6.943	0.949
	HPF	9.382	11.105	4.390	15.068	0.912
	PC	5.099	9.297	3.631	8.551	0.924
	Brovey	5.016	5.058	3.487	3.029	0.955
	Wavelet	9.118	11.120	4.513	12.131	0.981
	HCS	9.369	11.347	4.326	8.053	0.990
Band 2	Original MS	13.964	13.654	4.719	6.657	
	Modified-IHS	13.642	13.360	4.680	9.163	0.951
	HPF	13.472	13.639	4.769	18.586	0.912
	PC	7.903	11.523	4.366	11.576	0.921
	Brovey	7.137	6.185	3.857	3.811	0.954
	Wavelet	13.124	13.681	4.925	15.506	0.981
	HCS	13.470	13.950	4.767	10.075	0.989
Band 3	Original MS	18.772	22.142	5.205	9.984	
	Modified-IHS	18.278	21.817	5.230	13.703	0.964
	HPF	18.287	21.931	5.350	29.649	0.918
	PC	9.731	17.961	4.041	16.247	0.919
	Brovey	9.571	10.280	4.359	5.664	0.966
	Wavelet	17.778	22.086	5.415	24.162	0.981
	HCS	18.298	22.511	5.254	14.391	0.992
Band 4	Original MS	73.219	19.295	6.103	14.345	
	Modified-IHS	73.751	21.862	6.241	25.726	0.855
	HPF	72.708	19.167	6.088	28.634	0.907
	PC	78.718	15.430	5.807	18.014	0.968
	Brovey	37.537	7.950	4.380	9.656	0.854
	Wavelet	73.079	19.404	6.107	20.581	0.984
	HCS	72.718	19.733	6.171	22.856	0.981

**Table 3:** The average absolute error of 6 types of fused images in each band.

	Band 1	Band 2	Band 3	Band 4
Modified-IHS	1.647	2.241	3.086	8.247
Brovey	4.860	6.838	9.224	35.739
HCS	1.045	1.260	1.698	2.867
HPF	2.493	3.090	5.051	5.261
PC	4.936	6.283	9.439	5.864
wavelet	1.875	2.332	3.733	2.340

**Table 4:** Number of objects in 6 fused images.

	Brovey	HCS	HPF	Modified-IHS	PC	Wavelet
Number of objects	297	777	930	737	738	653

**Table 5:** Overall classification accuracy and Kappa coefficient of 6 types of fused images.

	Brovey	HCS	HPF	Modified-IHS	PC	Wavelet
OA	0.653	0.811	0.842	0.723	0.679	0.701
Kappa	0.447	0.665	0.720	0.504	0.458	0.505

## Data Availability

Data available on request from the authors.
